# Antifungal defense of probiotic *Lactobacillus rhamnosus* GG is mediated by blocking adhesion and nutrient depletion

**DOI:** 10.1371/journal.pone.0184438

**Published:** 2017-10-12

**Authors:** Daniela Mailänder-Sánchez, Christina Braunsdorf, Christian Grumaz, Christoph Müller, Stefan Lorenz, Philip Stevens, Jeanette Wagener, Betty Hebecker, Bernhard Hube, Franz Bracher, Kai Sohn, Martin Schaller

**Affiliations:** 1 Department of Dermatology, University Hospital Tübingen, Germany; 2 Fraunhofer IGB, Stuttgart, Germany; 3 Department of Pharmacy, Center for Drug Research, Ludwig-Maximilians University, Munich, Germany; 4 Center for Integrative Bioinformatics Vienna, Max F. Perutz Laboratories, University of Vienna, Medical University of Vienna, Vienna, Austria; 5 IGVP, University of Stuttgart, Stuttgart, Germany; 6 Department of Microbial Pathogenicity Mechanisms, Leibniz Institute for Natural Product Research and Infection Biology–Hans Knoell Institute Jena (HKI), Jena, Germany; 7 Center for Sepsis Control and Care (CSCC), Jena, Germany; 8 Friedrich Schiller University, Jena, Germany; King's College London Dental Institute, UNITED KINGDOM

## Abstract

*Candida albicans* is an inhabitant of mucosal surfaces in healthy individuals but also the most common cause of fungal nosocomial blood stream infections, associated with high morbidity and mortality. As such life-threatening infections often disseminate from superficial mucosal infections we aimed to study the use of probiotic *Lactobacillus rhamnosus* GG (LGG) in prevention of mucosal *C*. *albicans* infections. Here, we demonstrate that LGG protects oral epithelial tissue from damage caused by *C*. *albicans* in our *in vitro* model of oral candidiasis. Furthermore, we provide insights into the mechanisms behind this protection and dissect direct and indirect effects of LGG on *C*. *albicans* pathogenicity. *C*. *albicans* viability was not affected by LGG. Instead, transcriptional profiling using RNA-Seq indicated dramatic metabolic reprogramming of *C*. *albicans*. Additionally, LGG had a significant impact on major virulence attributes, including adhesion, invasion, and hyphal extension, whose reduction, consequently, prevented epithelial damage. This was accompanied by glucose depletion and repression of ergosterol synthesis, caused by LGG, but also due to blocked adhesion sites. Therefore, LGG protects oral epithelia against *C*. *albicans* infection by preventing fungal adhesion, invasion and damage, driven, at least in parts, by metabolic reprogramming due to nutrient limitation caused by LGG.

## Introduction

As commensal microbe, the yeast *Candida albicans* is a common inhabitant of various mucosal surfaces of the human body. However, under opportune conditions, *C*. *albicans* can transform from an innocuous commensal to an aggressive pathogen, causing relatively harmless superficial infections but also life threatening sepsis. Predisposing factors for the development of localized *Candida* infections are mainly immunosuppression [1] and an imbalance of the autochthonous microflora after antibiotic treatment [2], while epithelial barrier breach and neutropenia are the most common predisposing conditions for systemic infections [3]. In recent years, the human microflora has gained increased scientific interest, as its importance for maintaining human health has become more and more evident, reviewed recently by Lin et al. [4]. One concept evolved from the ongoing research in this field includes the use of probiotic microorganisms to influence the resident microbiota and/or the host immune system to prevent or cure diseases. A number of trials show a positive effect of probiotics in the treatment of various diseases and disorders, e.g. atopic dermatitis [5], irritable bowel syndrome [6, 7], ulcerative colitis [8] and antibiotic-associated diarrhea[9]. Even preterm neonates are medicated with probiotics to prevent infections [10–12]. The use of probiotic microorganisms to protect mucosal surfaces from *C*. *albicans* infections was recently reviewed by our group [13]. Several clinical trials indicate a protective role for probiotics in the prevention of superficial *C*. *albicans* infections, and diverse strains of the genus *Lactobacillus* have been found to be useful for the prevention and treatment of vulvovaginal candidiasis [14, 15], while combinations of different genera improved the outcome of oral candidiasis [16, 17]. Previous studies addressing the mechanisms behind probiotic action on *C*. *albicans* explored the ability of probiotic bacteria to inhibit fungal growth [18–21], to prevent adhesion [22, 23] or to influence anti-fungal immune responses [23, 24], respectively. Despite the numerous studies on probiotics, the mechanisms behind the beneficial effects of probiotics on human health and well-being are still unclear. A better understanding of these protective mechanisms would help to improve the use of probiotics in therapy and prevention of candidiasis. *Lactobacillus rhamnosus* GG (LGG) is a probiotic strain used for example in the treatment of diarrhea in children [25]. It has been successfully used to prevent colonization of mucosal surfaces with *C*. *albicans* in humans [11] and its safety has recently been confirmed by Manzoni and colleagues [26]. Here, we aimed to unravel the molecular mechanisms underlying the protective effect of LGG against *C*. *albicans* using an *in vitro* model of oral candidiasis.

## Materials and methods

### Microbial strains and growth conditions

*Candida albicans* SC5314 and *Lactobacillus rhamnosus* GG (LGG; InfectoPharm, Germany) were used in this study. LGG was grown for 36 h on MRS agar plates (Oxoid Deutschland GmbH, Germany) at 37°C and 5% CO_2_. Prior to inoculation LGG was washed once in PBS and diluted to an optical density OD_600_ of 0.669 (this equals McFarland standard No. 4). 10 ml of this suspension were centrifuged at 5000 rpm for 20 min and resuspended in 2 ml PBS. An aliquot of this suspension was then used for the experiments. *C*. *albicans* was grown on Sabouraud´s dextrose agar (BD Difco™, Beckton, Dickinson and Company, USA) followed by two precultures of a starting inoculum of 5 x 10^6^/ml in 10 ml YPD (1% yeast extract, 2% peptone, 2% dextrose) for 16 h at 25°C and further 24 h at 37°C as described previously [27]. Prior to infection, yeast cells were washed three times and diluted to the desired concentration with PBS.

### Cell culture and generation of three-dimensional mucosal models

TR146 cells (SkinEthic, France) [28] were cultured in D-MEM with 10% fetal bovine serum (FBS; Lonza, Switzerland) and 0.1% gentamicin solution (Sigma-Aldrich, Germany; 50 mg/ml) at 37°C and 5% CO_2_. For monolayer experiments, cells were seeded in KGM-Gold™ medium without antibiotics (Lonza, Switzerland). For invasion-assays an acetic acid-treated coverslip (15 mm diameter) was added to each well of a 24-well plate prior to cell seeding. For generation of three-dimensional RHOE TR146 cells were cultivated for 8 days in filter inserts, until they formed a multilayered epithelium (as detailed in supplementary data, text and S1 Table, and [29]).

### Infection of RHOEs and TR146 cell monolayers

RHOEs or TR146 cell monolayers, respectively, were preincubated with LGG for 12 h prior to infection with *C*. *albicans*. For the exact amount of TR146 cells, LGG and *C*. *albicans* used in the respective experiments please see Table 1. RHOEs were infected for 24 h, medium was changed at t = 12 h and t = 24 h. TR146 cell monolayers were infected for 1 h (adhesion), 3 h (invasion and hyphal growth), 2 h (RNA-Seq) and 6 h (RNA-Seq, damage, ergosterol quantification), respectively.

**Table 1:** CFU of LGG and *C*. *albicans* used for different experiments.

Experiment	Format	Medium	No of TR146 cells	CFU of LGG	CFU of *C*. *albicans*
**Infection of RHOE**	6-well plate	1 ml per well	n/a	3.8 x 10^6^ per RHOE	1 x 10^5^ per RHOE
**Infection of monolayers**	24-well plate	2 ml per well	2 x 10^5^ per well	1.9 x 10^6^ per ml	5 x 10^5^ per ml
**FUN-1 assay**	15 ml tube	5 ml per tube	n/a	3.8 x 10^6^ per ml	2 x 10^6^ per ml
**TEM analysis**	15 ml tube	10 ml per tube	n/a	1.9 x 10^6^ per ml	1 x 10^6^ per ml
**WST-1 assay**	15 ml tube / 96-well plate	6 ml / 100 μl per tube / well	n/a	1.9 x 10^6^ per ml	5 x 10^5^ per ml
**LGG growth**	15 ml tube	8 ml per tube	n/a	1.9 x 10^6^ per ml	n/a
***C*. *albicans* growth**	15 ml tube	8 ml per tube	n/a	n/a	1 x 10^6^ per ml
**Adhesion**	96-well plate	200 μl per well	3 x 10^4^ per well	1.9 x 10^6^ per ml	5 x 10^5^ per ml
**Invasion + hyphal growth**	24-well plate	2 ml per well	2 x 10^5^ per well	1.9 x 10^6^ per ml	5 x 10^5^ per ml

Numbers of human keratinocytes (TR146 cells) and colony forming units (CFU) of LGG and *C*. *albicans* used in the different experiments. Please note that adhesion assay equals one-tenth of monolayer and WST-1 assay one-twentieth, respectively.

### Epithelial cell damage

To analyze the damage of the epithelial cells caused by *C*. *albicans*, release of lactate dehydrogenase (LDH) into the supernatant of TR146 monolayers and RHOEs was quantified, using the cytotoxicity detection kit with L-LDH solution as standard, according to the manufacturer´s instructions (Roche, Germany). Supernatants of RHOEs were analyzed 24 h post infection, supernatants of monolayers 2 h and 6 h post infection.

### Effect of factors released by TR146 cells, *C*. *albicans* and LGG on the activity of LDH enzyme

To determine, whether TR146 cells, *C*. *albicans* or LGG do release factors that affect the activity of the LDH enzyme supernatants of one multilayer and one monolayer experiment were supplemented with 275 U/L L-LDH (Roche, Germany) and incubated for 1 h at 37°C and 5% CO_2_. An untreated control for each tested samples was run in parallel as a background control. LDH activity was quantified as _A492-620nm_ using the substrate provided with the cytotoxicity detection kit (Roche, Germany) in 1:2 diluted samples. Activity of untreated controls was subtracted to correct the background LDH activity.

### Yeast viability

To determine *C*. *albicans* viability in the presence or absence of LGG FUN-1 staining kit (Molecular Probes^®^, Thermo Fisher Scientific, Germany) was used. KGM-Gold™ medium, without antibiotics, was preincubated with LGG for 12 h. Next, *C*. *albicans* was added and incubated for 24 h. Then FUN1-staining solution (10 μM) was added and incubated for 30 min at 30°C and 100 rpm in an orbital shaker. Yeasts were centrifuged and resuspended in 100 μl PBS for analysis. Samples were analyzed with a confocal laser scanning microscope (Leica TCS SP; Leica Microsystems) at × 630 magnifications.

### Light microscopy and transmission electron microscopy

Co-cultures of LGG and *C*. *albicans* were harvested 24 h post infection by centrifugation. Pellets were resuspended in Karnovsky fixative. RHOEs were fixed by placing them into 24-well plates prepared with 400 μl Karnovsky fixative per well and adding 400 μl Karnovsky fixative into the insert. Harvested co-cultures and RHOEs were prepared for microscopic analysis as detailed in the supplementary. Semi-thin sections (0.5 μm) for light microscopy were stained with Toluidin blue and examined with a Nikon Eclipse 80i –light microscope. Images were processed with a Nikon Digital Sight DS-Fi2 camera [30]. Ultra-thin sections for transmission electron microscopy (TEM) were handled as described in the supplements.

### Yeast metabolic activity

To assess the metabolic activity of *C*. *albicans* we used the WST-1 Cell Proliferation Reagent (Roche, Germany). KGM-Gold™ medium was preincubated for 12 h with LGG or PBS. 100 μl aliquots of these media were transferred to a 96-well plate, inoculated with *C*. *albicans* and incubated for 24 h. Medium containing PBS and LGG alone were used as controls. After addition of 10 μl WST-1 reagent cells were incubated for 1 h at 37°C, absorbance was read at 450 nm with a wavelength correction set at 620 nm.

### Determination of pH

pH of differentially treated media was determined using pH-Fix test strips (Machery Nagel, Germany) covering the range between pH 2.0 and 9.0.

### Enzyme-linked immunosorbent assay

Interleukin (IL) 8, granulocyte macrophage colony-stimulating factor (GM-CSF) and IL1α were quantified in the supernatants of RHOEs and TR146-monolayers using DuoSet ELISA-Kits (RnD Systems, US) according to the manufacturer`s instructions.

### Adhesion assay

TR146 cells were preincubated with LGG for 12 h. Then cells were infected with *C*. *albicans*. To quantify adhesion of *C*. *albicans* to epithelial cells 1 h post infection immunostaining and subsequent imaging with Li-cor system was performed, according to an adapted protocol for on-cell Western assays (Li-cor, USA). Read-out was performed with a Li-cor Odyssey SA imaging system using the automatic grid alignment setting for 96-well plates, resolution was set to 100 μm and the focus offset to 3.0 mm. See supplementary information for a more detailed protocol.

### Invasion assay and hyphal growth

To determine invasiveness of *C*. *albicans* we performed a two-step immunofluorescence staining adapted from Wächtler et al [30]. Invasiveness and hyphal growth was analyzed 3 h post infection. See supplementary data for further instructions. Microscopy was performed using a Zeiss Imager Z1 ApoTome microscope equipped with Zeiss Axiocam digital camera and Zeiss Axiovision 4.7 software (Zeiss, Germany). Approximately 70–120 *Candida* cells were analyzed for invasiveness to epithelial cells. To quantify hyphal growth of *C*. *albicans* red fluorescence (entire *Candida* cell) was used. The length of 70–100 hyphae was measured using the measuring tool of Adobe Photoshop (CS2).

### RNA extraction

Samples of TR146 monolayers for RNA-Seq analysis were collected 2 h and 6 h after infection with *Candida*. Cells were scrapped into 1 ml chilled PBS and frozen as beads by dropping this suspension into liquid nitrogen. Disruption was carried out using a Mixer Mill MM 200 (RETSCH, Germany) with a shaking frequency of 30/s under cryo conditions. The resulting powder was resuspended in lysis buffer RLTplus (QIAGEN, Germany), supplemented with 0.01% v/v of ß-mercaptoethanol. The extraction of total RNA was performed according to QIAGEN’s Mechanical Disruption Protocol for the isolation of total RNA from yeast, using the RNeasy Plus Mini Kit. The experiments were performed in triplicates. RNA quality and quantity was evaluated using an Agilent 2100 Bioanalyzer with RNA 6000 Nano Chips, following the manufacturer’s protocol.

### cDNA library preparation for Illumina sequencing

cDNA libraries were prepared using an initial amount of 500 ng of total RNA and Illumina’s TruSeq^TM^ RNA Sample Preparation v2 protocol. Sequencing was performed using an Illumina HiSeq^TM^ 2000. To assess concentration and ensure an appropriate size distribution (between 200–570 bp) the cDNA libraries were checked using Bioanalyzer DNA 1000 chips. Sequencing run was carried out with single-end 50 bases long reads following the manufacturer’s instructions.

### Mapping and quantification of RNA-Seq data

Mapping was performed with NextGenMap (v 0.4.12) using default settings [31]. We used sequence files from Assembly21 (http://www.candidagenome.org/download/sequence/Assembly21) and annotation files from Grumaz et al. [32] as reference database. Gene quantification was calculated with a python script `rpkmforgenes.py´ from the Sandberg laboratory (http://sandberg.cmb.ki.se/rnaseq) at readcount and RPKM level (= reads per kilobase of exon model per million mapped reads, according to Mortazavi et al. [33]) using uniquely mapped reads. However, differential gene expression profiling was carried out exclusively based on readcount quantification via edgeR package (version 3.4.2) developed for RNA-Seq data [34]. In order to minimize false positives, we applied stringent criteria considering genes with an adjusted p-value (FDR) < 0.001 and a log_2_ fold change ≤ -1.5 or ≥ 1.5 as being significantly differentially regulated between two conditions. Sequence reads and quantification files generated from this study have been deposited in the Gene Expression Omnibus (GEO) under accession number GSE97755.

### Quantification of glucose in cell culture supernatant

Glucose was quantified in supernatants using the Glucose (GO) Assay Kit (Sigma Aldrich, Germany). Reagents were prepared according to the manufacturer`s instructions, samples were heat-inactivated at 95°C for 10 min. 50 μl per sample, standard or blank (H_2_O) were pipetted into a microplate, subsequently 100 μl Assay Reagent were added to each well. After 30 min incubation at 37°C the reaction was stopped by adding 100 μl 6 M H_2_SO_4_. Optical density was measured at 540 nm.

### Ergosterol isolation and quantification

To analyze ergosterol content of *C*. *albicans* cells, samples were harvested 6 h post infection. Pools of 12 wells (24-well plates) were collected in duplicates by scrapping them in 1 ml supernatant. After one wash in 2 ml PBS samples were disperged in 1 ml 2 M NaOH and sonicated for 5 min. After heating the samples for 90 min at 70°C, 5α-cholestane (Sigma Aldrich, Germany) (10 μg/ml in *tert*-butyl methylether (MTBE)) was added as internal standard (IS). Sterols (ergosterol from *C*. *albicans* and cholesterol from TR146 cells) were extracted in two steps by micro-liquid-liquid extraction with MTBE (Carl Roth, Germany). The extracts were purified with dispersive solid phase extraction (Na_2_SO_4_:PSA, 7:1), Na_2_SO_4_ (Carl Roth) and PSA (Agilent, USA), and 1 ml of each sample was evaporated over night at room temperature before GC-MS analysis. The samples were reconstituted with 900 μl MTBE and 100 μl silylation mixture of MSTFA:TSIM (9:1) (Machery Nagel, Germany). The GC-MS was operated as described by Müller et al. [35]. For quantification the peak areas of the base peaks for 5α-cholestane (IS) *m/z* 217, cholesterol *m/z* 368, and ergosterol *m/z* 363 were used.

### Statistical analysis

Results are presented as means ± SEM from at least three independent experiments. Statistical analysis was performed using GraphPad Prism 4.03. For comparison of two groups two-tailed student´s t-test was performed, comparison of more than two groups was done using one-way analysis of variance (ANOVA) with the Bonferroni-method as post-test. Significance was set for p<0.05.

## Results

### *Lactobacillus rhamnosus* GG protects oral epithelial tissue from damage and modifies *C*. *albicans* induced immune response

**Fig 1:**
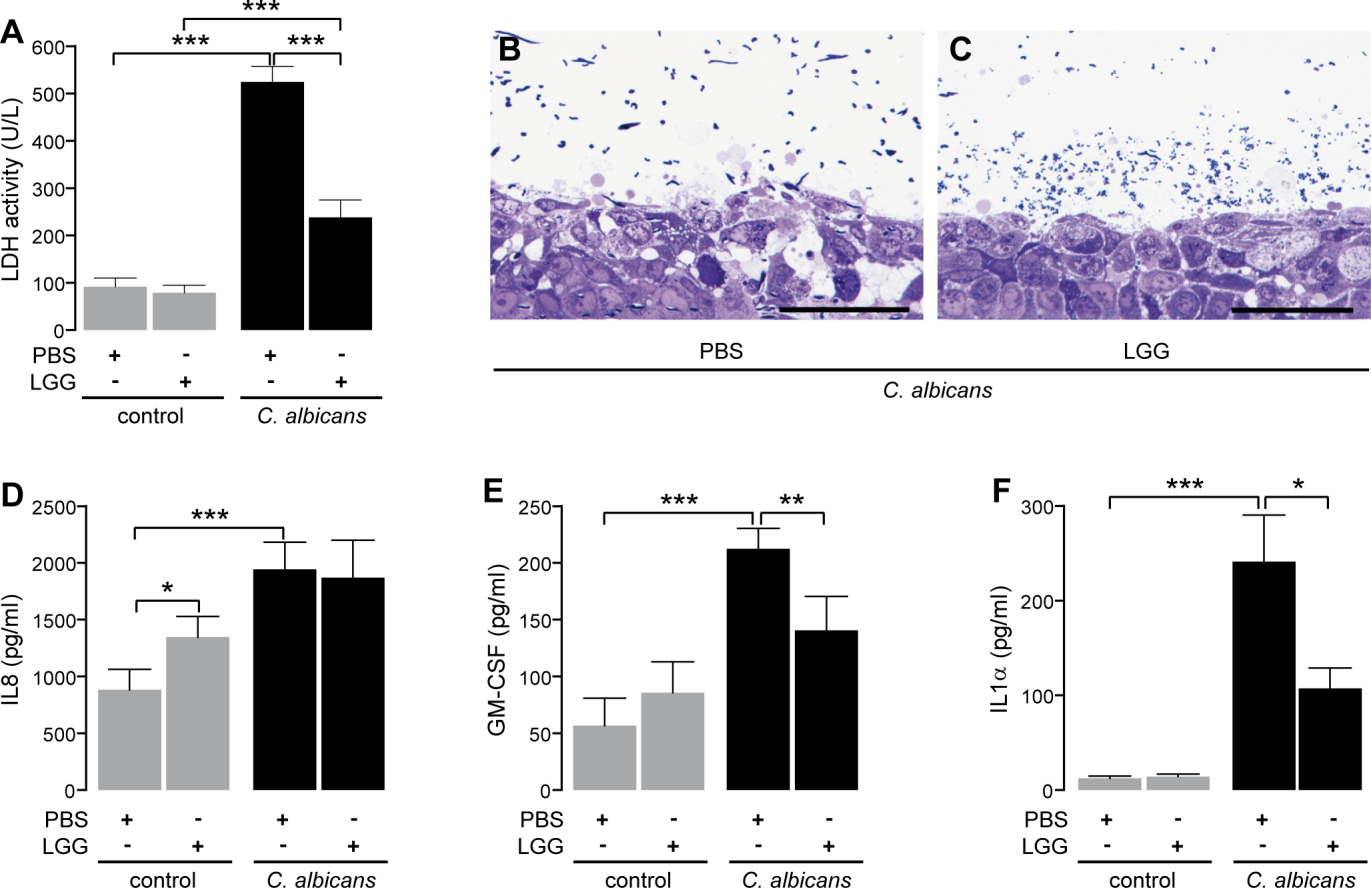
*L*. *rhamnosus* GG (LGG) protects against *C*. *albicans* infection (adapted from [29]). (A) *C*. *albicans*-induced release of lactate dehydrogenase (LDH). Semi-thin sections of *C*. *albicans*-infected RHOEs pretreated either with PBS (B) or LGG (C). Images are representative for three individual experiments. Scale bars equal 100 μm. Supernatants of RHOEs were further analyzed for cytokines. Content of interleukin-8 (IL8; D), granulocyte macrophage colony-stimulating factor (GM-CSF; E) and IL1α (F) was quantified. n = 3–7 *p<0.05, **p<0.01, ***p<0.001.

To investigate whether *Lactobacillus rhamnosus* GG (LGG) provides protection against an infection with *C*. *albicans* we used a multilayer of oral epithelial cells (Reconstructed Human Oral Epithelium–RHOE). RHOEs were preincubated with LGG for 12 h followed by infection with *C*. *albicans* for 24 h. Damage of RHOEs was quantified by measurement of levels of epithelial lactate dehydrogenase (LDH) released into the RHOE supernatant. As shown in Fig 1A, LGG alone did not induce damage and consequently release of LDH, while infection with *C*. *albicans* resulted in significant release of LDH, indicating lysis of epithelial cells. Preincubation with LGG lead to a strong reduction of *C*. *albicans*-induced LDH release, providing a first clear evidence for a protective effect of LGG. Still, RHOEs infected with *C*. *albicans* and treated with LGG showed a significantly higher release of LDH than the uninfected controls. However, histological analyses confirmed the protective effect of LGG. While *C*. *albicans* cells invaded the whole epithelium and caused vacuoles as signs of damage in absence of LGG (Fig 1B) RHOEs preincubated with LGG showed strongly reduced signs of damage and only in the uppermost cell layer (Fig 1C). In order to rule out a direct inhibitory effect of factors secreted by either TR146 cells, *C*. *albicans* or LGG on the activity of LDH enzyme, we added L-LDH to the supernatants obtained from one experiment. As shown in Table 2 (upper panel), the activity of the enzyme did not show differences, regardless of the used supernatant. Probiotics are described to modulate inflammation, as reviewed by Plaza-Diaz et al. [36]. Therefore, we questioned whether LGG has an impact on the inflammatory response of infected RHOEs. Hence, supernatants of RHOEs were tested for cytokine release by ELISA. As shown in Fig 1D–1F, *C*. *albicans* alone induced a significant release of IL8, GM-CSF and IL1α. Preincubation of RHOEs with LGG significantly reduced the *C*. *albicans*-induced release of GM-CSF and IL1α but interestingly not IL8 (Fig 1D–1F). LGG alone failed to induce GM-CSF and IL1α, but slightly activated IL8 release. Of note, treatment of RHOEs with LGG did not change the pH of the RHOEs supernatants (pH 7.5 in controls and LGG-treated samples). This is of importance, as the pH in the oral cavity is usually near neutral. Therefore, our model is suitable to mimic an oral *C*. *albicans* infection. Taken together, these data not only indicate a strong protective effect for LGG in our model of oral candidiasis but also a potential immunmodulation by this probiotic.

**Table 2:** Influence of experimental supernatants on LDH activity.

Model	Experimental condition	OD_492-620nm_
RHOE	PBS	1.95
	LRGG	1.95
	*C*. *albicans*	1.91
	*C*. *albicans* + LRGG	1.93
TR146 Monolayer	PBS	0.57
	LRGG	0.52
	*C*. *albicans*	0.59
	*C*. *albicans* + LRGG	0.55

### LGG does not directly inhibit fungal viability

**Fig 2:**
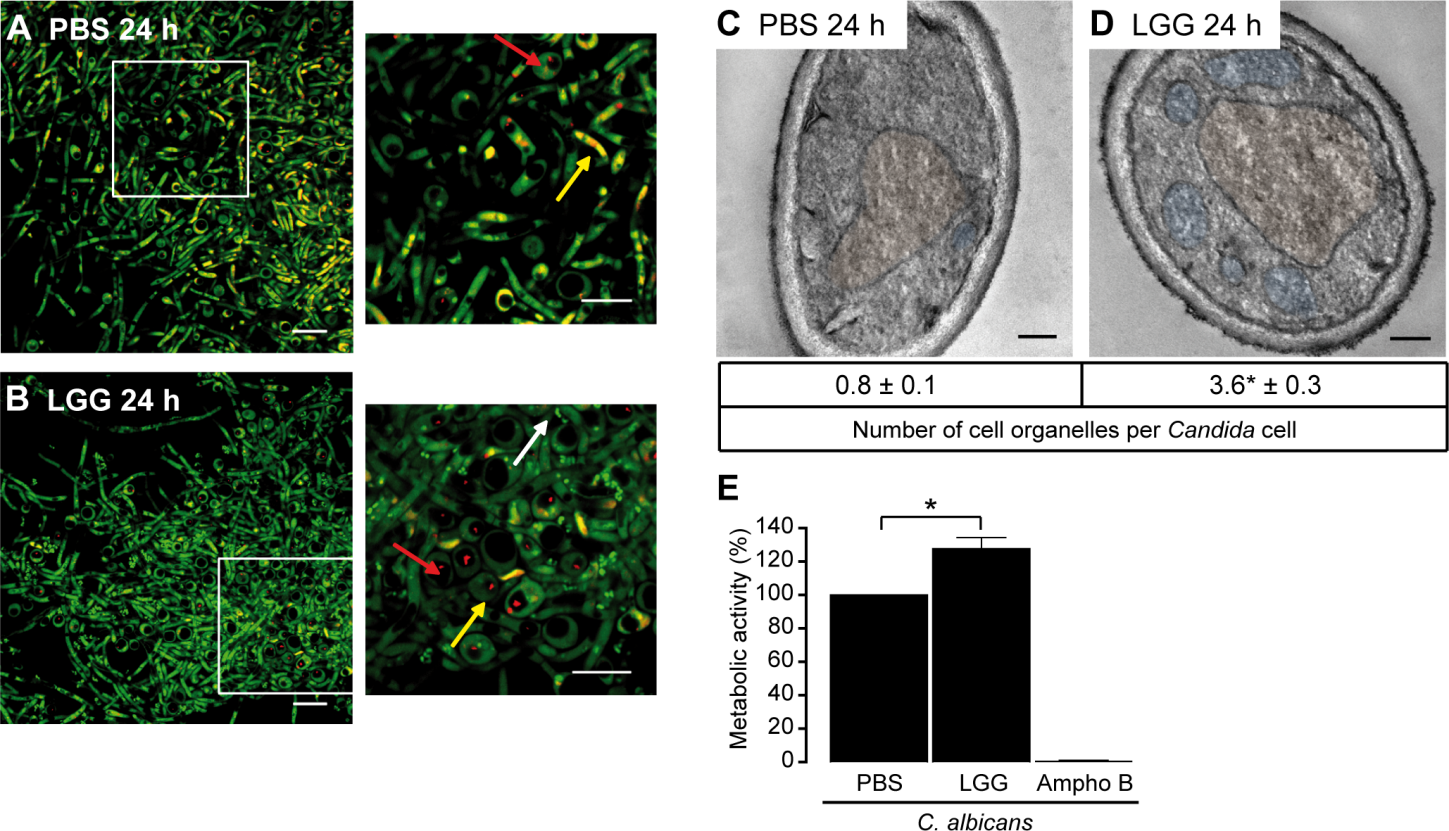
*C*. *albicans* viability in response to LGG. (A and B adapted from [29]). (A and B) FUN1-staining of *C*. *albicans*, grown in absence (A) or presence of LGG (B). Fluorescence staining was performed after 24 h of co-culture. Images are representative of three individual experiments. Scale bars equal 10 μm. Live fungal cells are indicated by green fluorescence and a well-defined vacuole structure. Red fluorescence inside these vacuoles indicates metabolic activity (red arrows). Yellowish fluorescence indicates dead fungi (yellow arrows). LGG stains green (white arrow). (C and D) Transmission electron microscopy of *C*. *albicans*. *C*. *albicans* was grown for 24 h in absence (C) or presence (D) of LGG. Cell organelles were counted in 25 cells per condition and experiment. Nuclei are highlighted in brown, organelles in blue. Micrographs are representative for three individual experiments. Scale bars equal 0.2 μm. (E) Metabolic activity of *C*. *albicans* grown in absence or presence of LGG, Amphotericin B (Ampho B; 0.1 μg/ml) served as control. (C, D and E) n = 3 *p<0.05.

To test whether LGG directly affects the viability of *C*. *albicans*, we performed several assays in the absence of epithelial cells. First, we used FUN1-Live/Dead-staining. Fungal cells were cultivated for 24 h in medium preincubated for 12 h with PBS or LGG. Results of fluorescence microscopy analysis are presented in Fig 2A and 2B. *C*. *albicans* grown in co-culture with LGG (Fig 2B), showed no impaired metabolic activity or increased amount of dead cells compared to *C*. *albicans* grown in absence of LGG (Fig 2A). Furthermore, no impact on hyphal growth was observed. As lactobacilli also stain green by FUN1-staining (see Fig 2B, white arrow) we did not perform additional quantification of fungal viability via fluorescence intensity. To further investigate a possible negative impact of LGG on *C*. *albicans* fitness, we used transmission electron microscopy (TEM). As shown in Fig 2 *C*. *albicans* cells grown in presence of LGG (Fig 2D) had an intact cell wall and showed no signs of structural disintegration compared to *C*. *albicans* grown in the absence of LGG (Fig 2C). Instead, *C*. *albicans* cells showed a dramatic reorganization of organelles, such as mitochondria and peroxisomes, indicating changes of metabolism. To verify this finding we used the WST-1 assay to quantify metabolic activity of *C*. *albicans*. As shown in Fig 2E co-incubation with LGG significantly increased the metabolic activity of *C*. *albicans*, while Amphotericin B, an antifungal drug, completely inhibited this activity. In summary, we found no evidence for a direct antifungal effect of LGG. Instead, our data point to a so far undescribed reorganization of *C*. *albicans* metabolism in response to LGG.

### Analysis of the effect of LGG on *C*. *albicans* gene expression by next generation sequencing

**Fig 3:**
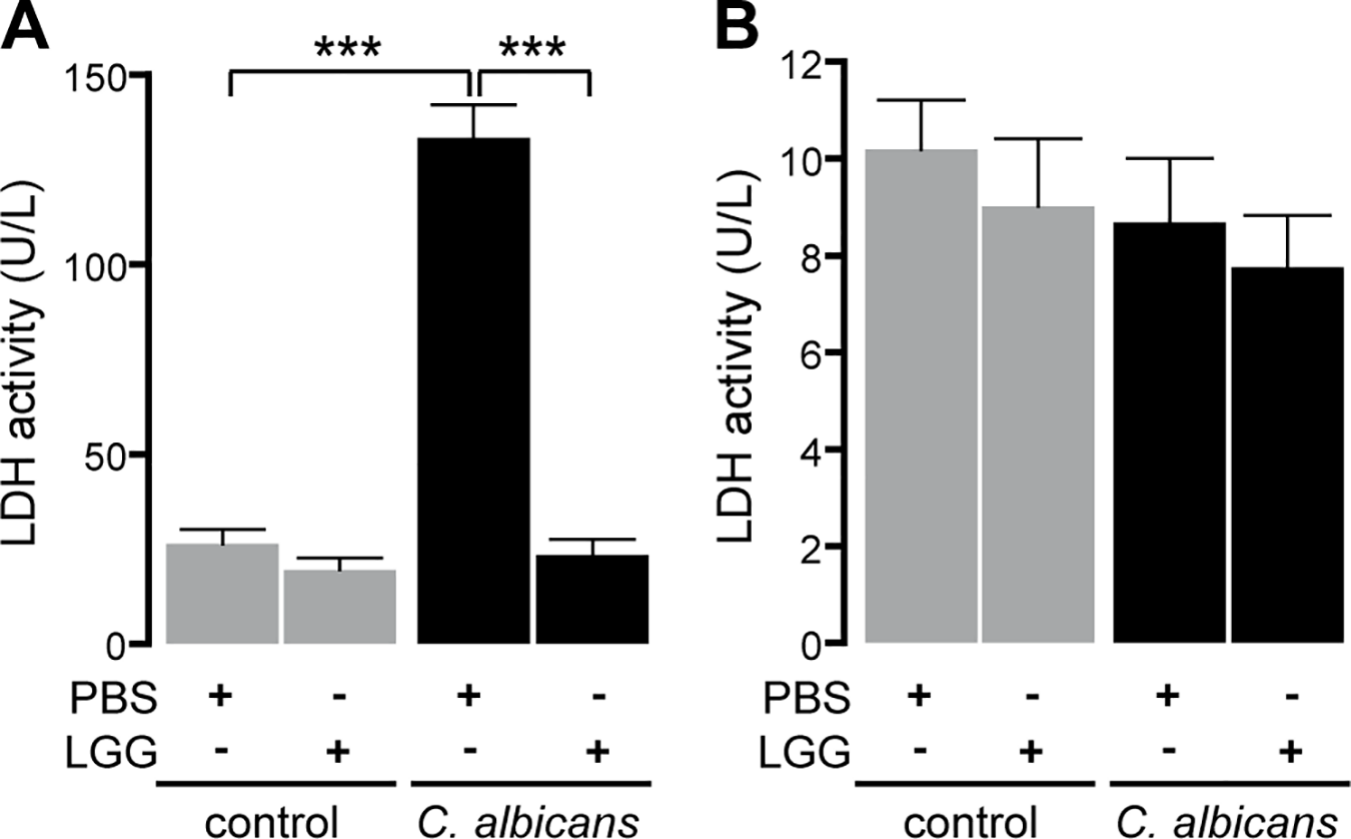
LGG mediates protection against *C*. *albicans* infection in TR146 monolayer. LDH activity in response to *C*. *albicans* infection was quantified in cell culture supernatant 6 h (A) and 2 h (B) post infection. n = 3 ***p<0.001.

**Fig 4:**
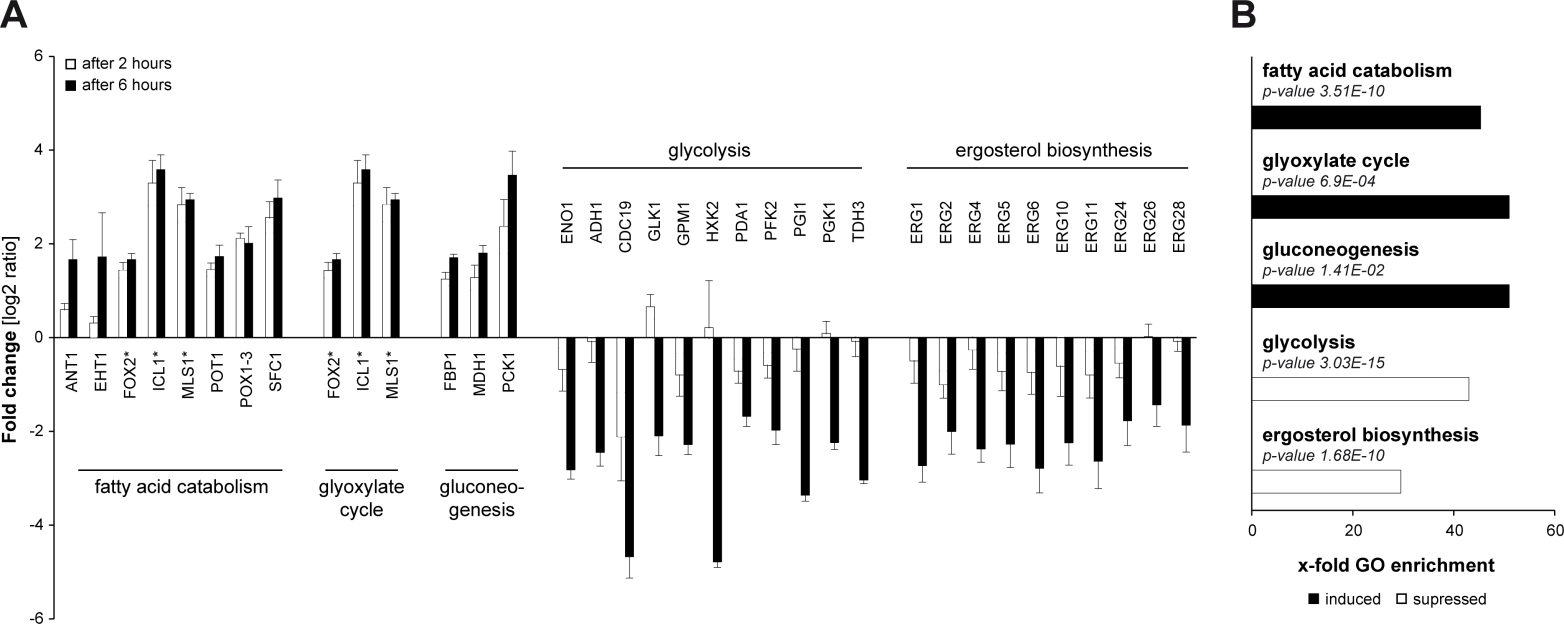
Regulation of *C*. *albicans* gene expression in response to LGG. Monolayers of TR146 cells were preincubated with PBS or LGG for 12 h. Then, epithelial cells were infected for 6 h with *C*. *albicans* cells. For analysis of mRNA expression this experiment was performed three times. (A) Fold changes of mRNA expression in *C*. *albicans* in response to LGG. (B) Gene ontology enrichment of metabolic pathways in *C*. *albicans* in response to LGG. Corresponding sets of up- and down-regulated genes were mapped with the „GO term finder” at CGD to biological processes. X-fold enrichment is calculated as ratio of percentages of the cluster frequency of tested gene set and the cluster frequency of genomic background. * *FOX2*, *ICL1* and *MLS1* are associated with fatty acid catabolism and glyoxylate cycle. n = 3.

As shown above, we found no evidence for a direct killing effect of LGG against *C*. *albicans* (Fig 2A, 2B and 2E). Instead, our results indicated metabolic changes in *C*. *albicans* cells due to the presence of LGG (Fig 2). Therefore, we analyzed the response of *C*. *albicans* to LGG-preincubated cells on the transcriptional level by RNA-Seq. As the samples extracted from infected RHOEs yielded unfavorable proportions of *C*. *albicans* RNA compared to the vast excess of co-isolated human RNA, we decided to isolate RNA from an equivalent monolayer model of oral epithelia cells (TR146) instead. For this reason, we first confirmed the protective effect of LGG preincubation in this system. TR146 monolayers were preincubated with LGG 12 h prior to *C*. *albicans* infection. Epithelial cells were subsequently infected with *C*. *albicans* cells and LDH activity was analyzed 6 h post infection. LGG also protected TR146 monolayers (Fig 3A), similar to what we had already observed in RHOEs. Again, we did not observe any direct inhibition of LDH activity, as summarized in Table 2 (lower panel). Additionally, we quantified the LDH release of TR146 monolayers 2 h after infection with *C*. *albicans*. At this time, no damage was detected (Fig 3B). Therefore, time points 2 h and 6 h post infection were chosen for gene expression analysis. In total, we identified 33 and 161 differentially regulated genes 2 h and 6 h post infection, respectively (S2 Table). Among these, genes coding for enzymes of five different metabolic pathways were significantly affected in *C*. *albicans* cells co-cultivated with LGG on TR146 cells as compared to *C*. *albicans* cells on PBS-pretreated controls (Fig 4A). Presence of LGG had significant effects on the induction of genes involved in fatty acid catabolism, glyoxylate cycle and gluconeogenesis (~40 to 50-fold) and on suppression of genes contributing to glycolysis and ergosterol biosynthesis (~30 to 40-fold) as revealed by gene ontology (GO) enrichment analysis of differentially expressed *C*. *albicans* genes (Fig 4B). These results indicated a lack of glucose as the major carbon source and a dramatic change in the composition of *C*. *albicans* cell membrane.

### Impact of LGG on *C*. *albicans* ergosterol content and glucose levels

**Fig 5:**
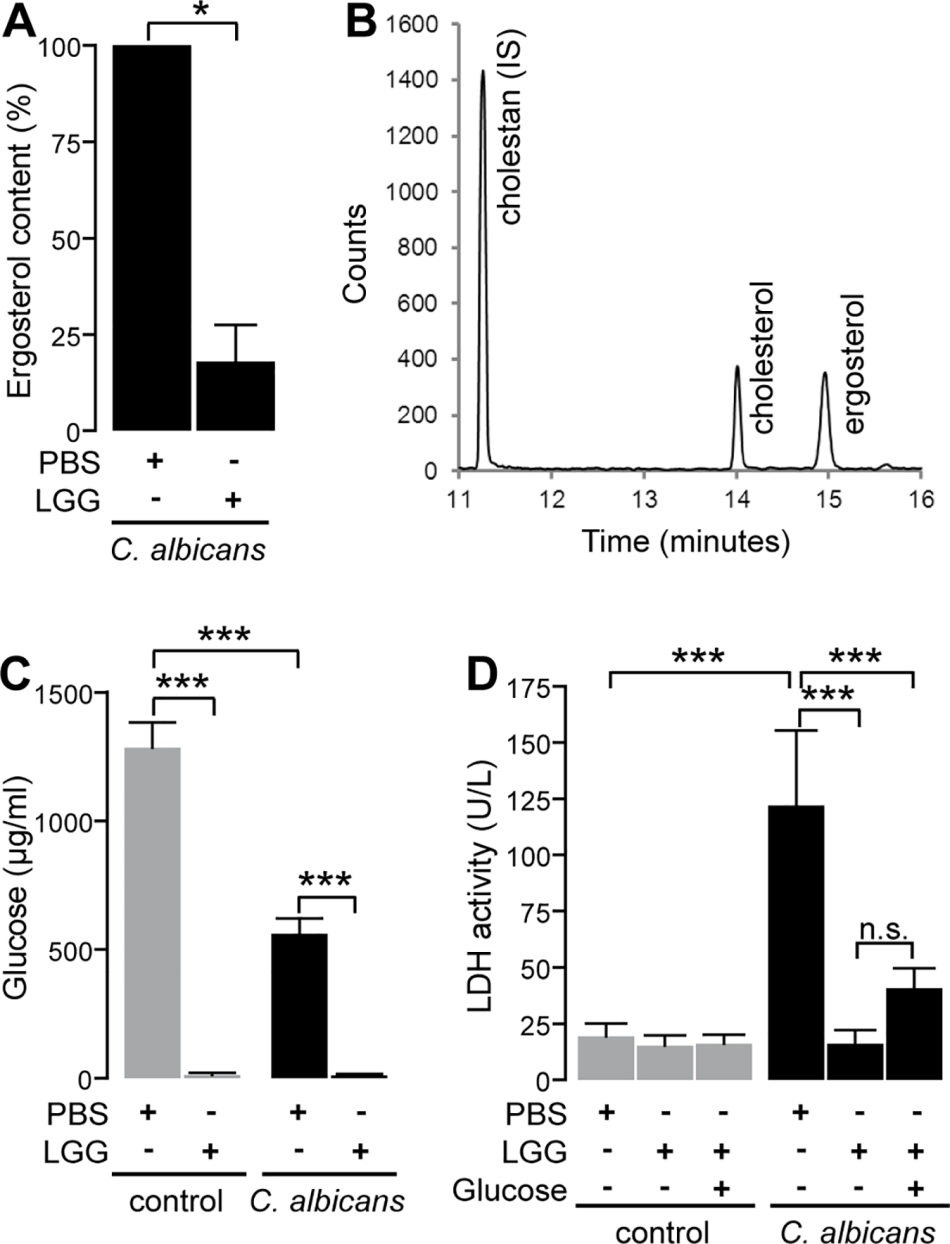
Impact of LGG on *C*. *albicans* ergosterol content and glucose concentration in cell culture supernatants. (A) Extracts of *C*. *albicans* grown on TR146 monolayers pretreated with either PBS or LGG were analyzed for their ergosterol content. (B) Exemplary selected ion chromatogram of one sample, where LGG-preincubated cells were infected with *C*. *albicans*, containing cholestane (*m/z* 217), cholesterol (*m/z* 368) from TR146 cells and ergosterol (*m/z* 363) from *C*. *albicans*. (C) Monolayers of TR146 cells were preincubated with PBS or LGG 12 h prior to infection with *C*. *albicans* cells. Amount of glucose was evaluated 6 h post infection. (D) Monolayers of TR146 cells were preincubated with PBS or LGG 12 h prior to infection. Directly before infecting epithelial cells with *C*. *albicans*, glucose was added to the experiment (5 mg/ml). LDH activity was quantified 6 h post infection. n = 3 *p<0.05, ***p<0.001.

Ergosterol is a major component of *C*. *albicans* cell membrane and required for hyphal growth [37]. As RNA-Seq data suggested down-regulation of genes involved in ergosterol synthesis in the presence of LGG, we determined the content of fungal ergosterol after derivatization to the trimethylsilyl ether by gas chromatography mass spectrometry (GC-MS). As shown in Fig 5A, LGG pretreatment significantly decreased the content of ergosterol in the cell membrane of *C*. *albicans* during infection. As the expression of enzymes contributing to glycolysis was also reduced in the presence of LGG, we next determined a possible reduction of glucose availability in the medium. Therefore, we quantified glucose levels in the supernatant of infected TR146 monolayers with and without LGG. As shown in Fig 5C, preincubation with LGG lead to an almost complete depletion of glucose, thereby depriving *C*. *albicans* of its major carbon source. Consequently, we addressed the question, whether the lack of this nutrient alone could lead to the observed protection by supplementing the medium with 5 mg/ml glucose before starting *C*. *albicans* infection. As seen in Fig 5D, addition of glucose had no significant effect on LGG mediated reduction of LDH release. As the added amount of glucose (5 mg/ml) is sufficient to maintain nutrient availability throughout the experiment (S3 Table, upper panel) we can conclude that nutrient depletion is not exclusively responsible for protection.

### Preincubation with LGG impairs major virulence attributes of *C*. *albicans*

**Fig 6:**
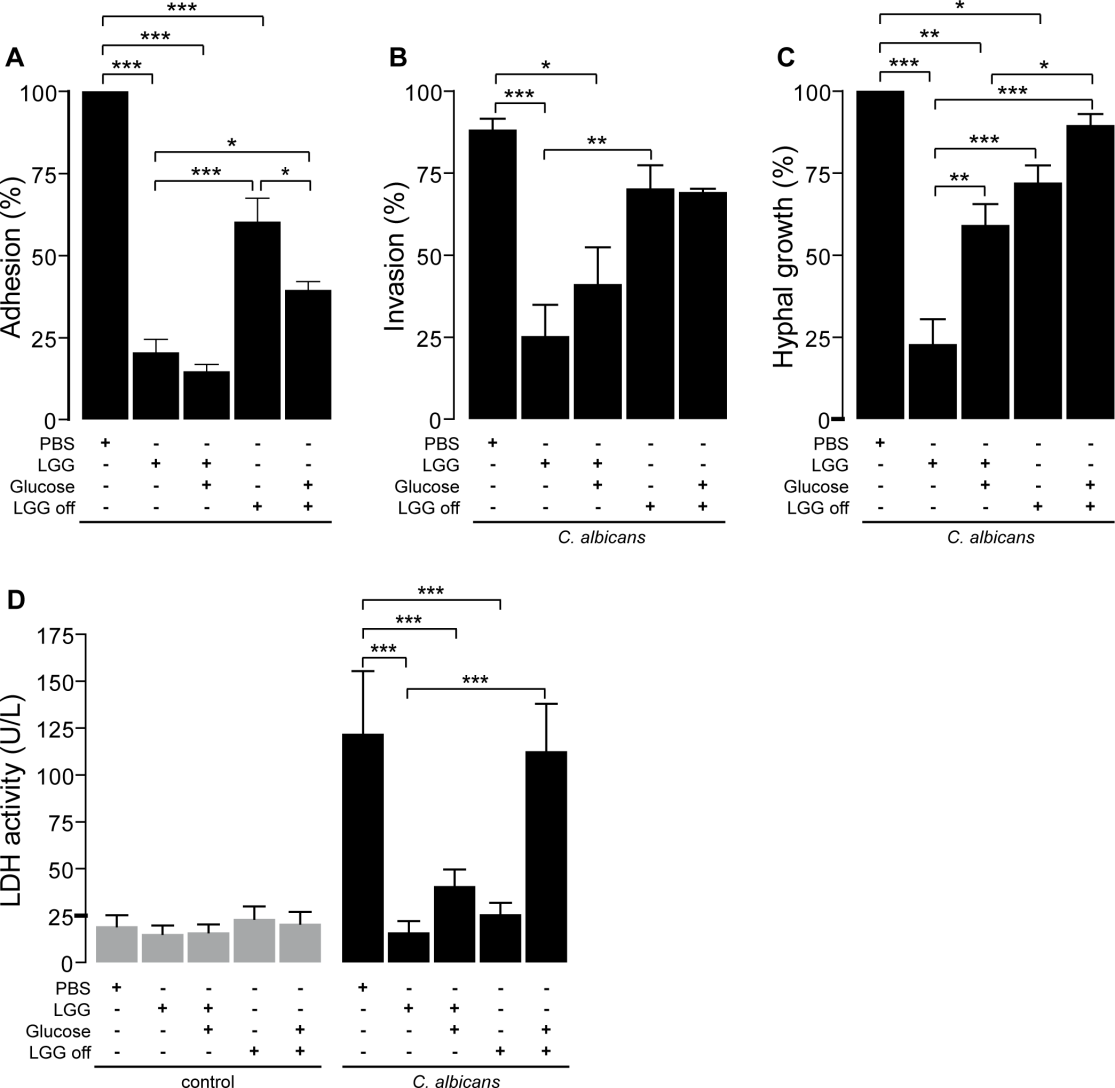
Preincubation of TR146 cells with LGG impairs major virulence attributes of *C*. *albicans*. (A-C) Monolayers of TR146 cells were preincubated with PBS or LGG for 12 h. In some conditions LGG was then removed by rinsing the cells with PBS (LGG off) and/or glucose was added to the medium (5 mg/ml). Then, epithelial cells were infected with *C*. *albicans* cells. (A) Adhesion of *C*. *albicans* to epithelial cells was analyzed 1 h post infection. Invasiveness (B) and hyphal growth (C) was measured 3 h post infection. (D) LDH release by TR146 cells was quantified 6 h post infection. (A-D) n = 3 *p<0.05, **p<0.01, ***p<0.001.

Adhesion, invasion and hyphal growth are major virulence attributes of *C*. *albicans* and adhesion to epithelial surfaces is the first step in infection. Therefore, we investigated the effect of LGG on adhesiveness of *C*. *albicans* to TR146 monolayers. Preincubation of cell monolayers with LGG lead to a significant reduction of *C*. *albicans* adhesion (Fig 6A). Furthermore, invasiveness and hyphal growth of fungal cells were assessed. As shown in Fig 6, preincubation with LGG leads to a significant decrease of *C*. *albicans* invasion (Fig 6B) and hyphal growth (Fig 6C). Supplementation of the medium with glucose had no effect on adhesion (Fig 6A) and invasion (Fig 6B), but instead significantly enhanced hyphal growth of *C*. *albicans* (Fig 6C). Removing LGG by rinsing the cells with PBS before infection significantly increased adhesion (Fig 6A), invasion (Fig 6B) and hyphal growth (Fig 6C) of *C*. *albicans*. Additional supplementation with glucose suppressed adhesion (Fig 6A) but not invasion (Fig 6B) and restored hyphal growth almost completely (Fig 6C), with a significant difference to only adding glucose (Fig 6C). Thus, we determined release of LDH 6 h post infection under identical conditions. Fig 6D clearly shows that release of LDH after *C*. *albicans* infection was not increased by addition of glucose or removal of LGG alone. Only the combination of both lead to elevated LDH release and restored the LDH activity to control levels. Taken together, our data indicate that protection of epithelial cells against damage caused by *C*. *albicans* is mediated by LGG via depletion of nutrients in combination with blocking adhesion sites for the pathogen. These conditions repress hyphal growth and consequently reduce invasiveness of *C*. *albicans*, thereby preventing gross damage of epithelial cells.

## Discussion

The results of our work suggest that the protective effect provided by LGG against *C*. *albicans*-caused epithelial damage is mediated by a synergy of reduced adhesion and glucose depletion. Depletion of glucose led to a shift in *C*. *albicans* metabolism, resulting in an impaired formation of hyphae and consequently reduced damage (Fig 1A). To our knowledge, we provide for the first time a potential mechanism, how probiotic lactobacilli protect oral epithelial cells against damage caused by *C*. *albicans in vitro*.

Preincubation of RHOEs with LGG protects from *C*. *albicans*-induced cell damage, characterized by decreased levels of LDH in cell supernatants, reduced histological lesions and invasion into the epithelial cells as well as a lower inflammatory response (Fig 1E and 1F). Still, RHOEs infected with *C*. *albicans* and treated with LGG release more LDH than the LGG treated control. This is in line with the histological analysis showing little damage in the uppermost cell layer of the epithelium. Possibly, a higher CFU of LGG or a second dose would increase the protective effect. Nevertheless, our results clearly support our hypothesis of a protective effect of LGG in oral *C*. *albicans* infections. The results of the LDH assay are in accordance with two other studies [38, 39] reporting protective action of *Lactobacillus* ssp. against cytotoxic effects. In order to rule out the possibility that LDH could be degraded by components released by either LRGG, *C*. *albicans* or the TR146 cells we added a defined amount of LDH to the supernatants of one RHOE and one monolayer experiment. As shown in Table 2, no differences could be observed between the activities of LDH in the different supernatants. Therefore, the reduced activity of LDH in LRGG treated, *C*. *albicans* infected RHOEs and monolayers can be considered to be the result of the protective effect of LRGG. Also in line with our results, Ribeiro et al. recently showed a protective effect of *L*. *rhamnosus* ATCC 9595 against *C*. *albicans* infection in the larvae of wax moths (*Galleria mellonella*) [40]. Furthermore, we observed elevated release of IL8 in RHOEs treated with LGG although LGG does not damage the epithelial cells (Fig 1D). This is of special interest, as IL8 is a potent chemoattractant for polymorphonuclear cells (PMNs) and reduced PMN function is associated with increased susceptibility to oral colonization and infection with *C*. *albicans* [41]. Therefore, future studies should address the potential of LGG and other probiotics to attract and activate PMNs in order to prevent mucosal fungal infections.

Analysis of yeast viability by FUN1-staining revealed no direct antifungal effect of LGG on *C*. *albicans* growth or metabolic activity in the absence of epithelial cells (Fig 2A and 2B). These findings are in contrast to Köhler et al. [20] who showed reduced viability of *C*. *albicans* in the presence of *L*. *rhamnosus* GR-1 and *L*. *reuteri* RC-14, also using the FUN1-staining kit. This effect was dependent on low pH and the presence of lactic acid, detected in the medium (MRS broth) used. Therefore, we analyzed the differences of KGM-Gold™ and MRS (S4 Table). We found that LGG as well as *C*. *albicans* show better growth in MRS. Furthermore, pH remained at 7.5 in KGM-Gold™ while LGG decreased pH of MRS. Similar results were observed in the supernatants of oral keratinocytes after incubation with LGG and/or *C*. *albicans* (S5 Table). This is of importance, as the pH in the human oral cavity is usually neutral, indicating the relevance of KGM-Gold™ in a model for oral candidiasis while the lower pH levels obtained using MRS broth rather mimic the low pH level in the vagina. Furthermore, it is known that different strains of lactobacilli differ in their properties. Transmission electron microscopy (TEM) analysis of *C*. *albicans* cells did not indicate a loss of viability or cell death, adding to our hypothesis that loss of *C*. *albicans* viability is not responsible for protection in our model (Fig 2C and 2D). Instead we found an increased number of fungal mitochondria and peroxisomes, indicating changes in *C*. *albicans* cell biology and potentially metabolism, rather than killing due to the presence of LGG. This hypothesis was further strengthened by the finding that the metabolic activity of *C*. *albicans* was increased in the presence of LGG, especially as the assay we used is dependent on the activity of mitochondria [42]. Analysis of the transcriptional profiles of *C*. *albicans* infecting oral epithelial cells clearly confirmed our previous results. We found evidence for the activation of fatty acid catabolism, glyoxylate cycle and gluconeogenesis in the presence of LGG while glycolysis was strongly suppressed (Fig 4). These results are again in contrast to the observations of Köhler et al. [20], who showed a reduced expression of key genes of gluconeogenesis, *MDH1* and *PCK1*, while *GLK4* (glycolysis) was induced when *C*. *albicans* was grown in the presence of lactobacilli. Besides the different media and lactobacilli strains used, the presence of human epithelial cells in our experiments provide an additional explanation for the discrepancies between our studies. In addition, we found an activation of genes coding for the carnitine acetetyltranferases *CTN1* and *CTN3* (compare S2 Table) highlighting the findings in our TEM pictures, that show increased numbers of mitochondria and peroxisomes (Fig 2C and 2D). Both, fatty acid catabolism and glyoxylate cycle take place in these compartments [43]. Additionally, induction of *CTN* expression was also shown by Köhler [20]. Ctn1 is located at the mitochondrial outer membrane, while Ctn3 is located at the membrane of peroxisomes [43]. The increased expression of *CTN1* and *CTN3* mRNA therefore provides additional evidence for the changes in *C*. *albicans* metabolism in response to LGG, as these transferases transport acetyl-CoA across the membranes of their respective compartment. Under glucose depletion, fatty acid catabolism provides acetyl-CoA molecules by degradation of fatty acids. Acetyl-CoA is further metabolized by the glyoxylate cycle (under low-sugar conditions) to generate succinate required in the TCA cycle or to provide precursors for the biosynthesis of amino acids or carbohydrates [44]. This happens via gluconeogenesis, a pathway which was induced in *C*. *albicans* cells infecting epithelial cells in the presence of LGG (Fig 4). In contrast, several genes encoding enzymes of glycolysis, including the key enzyme phosphofructokinase, were suppressed under the same condition. Together this indicates a dramatic reprogramming of *C*. *albicans* metabolism driven by the absence of the preferred carbon source glucose. The increased consumption of acetyl-CoA by the glyoxylate cycle and gluconeogenesis might provide an explanation for the observed suppression of the ergosterol biosynthesis in *C*. *albicans* grown on LGG-treated keratinocytes, as acetyl-CoA is also the precursor for ergosterol biosynthesis [45]. A suppression of *C*. *albicans* ergosterol biosynthetic gene expression in consequence to lactobacilli was also observed by Köhler et al. [20]. Martin et al. [37] showed that ergosterol accumulates in lipid rafts at the tips of *C*. *albicans* hyphae. Blocking of this accumulation suppressed filamentation similar to our results in glucose depleted medium caused by LGG. Though addition of glucose reverted hyphal growth to a certain degree, it alone could not restore *C*. *albicans* induced damage of human keratinocytes (Fig 5D). Interestingly, Oliveira et al. showed very recently that presence of *L*. *rhamnosus* ATCC 7469 increased the susceptibility of *C*. *albicans* against fluconazole, an antifungal drug which inhibits ergosterol biosynthesis [46]. Thus, future experiments should also address the impact of probiotics on the efficacy of antimycotics. Further experiments revealed a dramatic impact of LGG on major virulence attributes of *C*. *albicans*. Preincubation of keratinocytes with LGG reduced adhesion, invasion and hyphal growth of *C*. *albicans* significantly. Impairment of *C*. *albicans* adhesion in response to lactobacilli has previously been reported [22, 23], and Oliveira et al. [46] showed an impact of *L*. *rhamnosus* ATCC7469 on several virulence attributes such as proteinase and haemolytic activity, germ tube and biofilm formation. Of note, none of these studies did address the contribution of metabolism and availability of glucose. Interestingly, our findings show that nutrient depletion alone is not sufficient to provide complete protection, since supplementing glucose did only partially restore hyphal growth without increasing adhesion, invasion or LDH release, respectively (Fig 6). However, when we additionally removed LGG from the epithelial cells invasion, hyphal growth and LDH activity reached levels of untreated *C*. *albicans* infection. Of note, LGG alone, in absence of the keratinocytes, did not affect *C*. *albicans* hyphal growth. This might be explained by our finding that neither LGG alone nor combined incubation of LGG and *C*. *albicans* in KGM-Gold™ lead to a complete depletion of glucose (S3 Table, lower panel). Therefore, *C*. *albicans* finds sufficient glucose to form hypha. Noverr et al. [47] observed an influence of LGG on serum induced germ tube formation of *C*. *albicans*. This effect could be reproduced using butyric acid, which is a product of *Lactobacillus* metabolism [48]. Butyrate is the shortest fatty acid that can be used for fatty acid catabolism [49]. The use of butyrate instead of glucose as substrate could explain the strong induction of fatty acid catabolism in *C*. *albicans* infecting LGG-treated cells. In summary, our study provides the first mechanistic insights how probiotic treatment can affect pathogenicity of *C*. *albicans* by restricting the availability of an important nutrient and preventing adhesion of this pathogen, thereby sheltering epithelial cells from damage.

## Supporting information

S1 File Supplementary materials and methods. (DOC)

S1 Fig Three-dimensional mucosal model. Reconstructed human oral epithelium (RHOE) at day 8 of cultivation. (A) Semi-thin section stained with toluidine blue and visualized by light microscopy at a 400-fold magnification. (B) Ultra-thin sections were contrasted with osmium and visualized by transmission electron microscopy. Arrows indicate desmosomes. Scale bar equates 0.5 μm.
(TIF)

S1 Table Generation of three-dimensional mucosal models. Summary of the different media used to generate three dimensional models of human oral mucosa.
(DOC)

S2 Table Results of gene expression analysis in *Candida albicans* cultivated in epithelial cells in presence and absence of *Lactobacillus rhamnosus* GG. (A) Quantification of gene expression in *C*. *albicans* 2 h and 6 h post-infection. (B) Gene expression in *C*. *albicans* after 2 h of infection. (C) Gene expression in *C*. *albicans* after 6 h of infection.
(XLS)

S3 Table Glucose content in KGM-Gold™ medium after 18 h in presence or absence of keratinocytes. The upper panel shows the amount of glucose in the supernatants of differentially treated monolayers of TR146 keratinocytes. In some cases the medium was supplemented with glucose (5 mg/ml). The lower panel shows the amount of glucose in KGM-Gold™ medium incubated with LGG and or *C*. *albicans*. n = 3.
(DOC)

S4 Table Differences between KGM-Gold™ medium and MRS medium. LGG (1.9 x 10^6^ CFU/ml) and *C*. *albicans* (1 x 10^6^ CFU/ml) were inoculated to KGM-Gold™ or MRS broth. OD_600_ was determined immediately and after 12 h incubation. pH was determined after 12 h incubation. To assess hyphal growth *C*. *albicans* was allowed to grow for 18 h.
(DOC)

S5 Table pH in the supernatant of differentially treated oral keratinocytes. (DOC)
