# Supplementary material for: Antifungal defense of probiotic Lactobacillus rhamnosus GG is mediated by blocking adhesion and nutrient depletion
Source: PLoS One. 2017 Oct 12;12(10):e0184438. doi: 10.1371/journal.pone.0184438 (PMC5638248; doi:10.1371/journal.pone.0184438)
Supplement: S1 File — (DOC) [file pone.0184438.s001.doc]

**Supplementary Information**

**Generation of Three-dimensional Skin Models**

For generation of three-dimensional skin models (RHOE) 1x10^6^ TR146 cells in 400 µl D-MEM (Lonza, Switzerland) medium with 10% FBS were seeded onto filter inserts (Millipore, Millicell-PCF 0.4 µm, 12 mm diameter), placed in 24-well plates containing 400 µl KBM™ medium/well (Lonza, without antibiotics); day 0 (d0). Medium was changed daily according to supplementary Table 5. After airlifting the RHOEs at d5 inserts were placed in 6-well plates containing 1 ml Medium according to supplementary Table 5. RHOEs were used for experiments at d8. By this time, keratinocytes have formed a stratified multilayered epithelium (see supplementary Figure 1A) containing desmosomes (see supplementary Fig. 1B, see arrows), similar to oral mucosa.

**Transmission Electron Microscopy**

RHOEs were fixed by placing them into 24-well plates prepared with 400 µl Karnovsky fixative per well and adding 400 µl Karnovsky fixative into the insert and stored at 4°C for 4 days. Microbial cultures containing either *C. albicans* or a mixture of *C. albicans* with LGG were collected by centrifugation, washed once with prewarmed PBS, fixed with warm Karnovsky’s solution containing paraformaldehyde and glutaraldehyde for 1 hour at room temperature and stored at 4°C for 4 days. For electron microscopic analysis, the microbial cultures were first embedded in 3.5% agarose at 37°C, coagulated at room temperature and after cutting, approximately 1 mm sections were fixed again in Karnovsky’s solution. Post-fixation microbial cultures and RHOEs were based on 1.0% osmium tetroxide containing 1.5% K-ferrocyanide in 0.1 M cacodylate buffer for 2 hours. Samples were rinsed in distilled water, treated with 1% aqueous uranyl acetate and dehydrated in ascending series of alcohol dilutions to 100%. Dehydrated samples were infiltrated with propylene oxide, embedded in glycide ether, followed by sectioning. For tissue identification semi-thin sections were stained with toluidine blue. Selected regions of interest were ultrathin sectioned at 30 nm, mounted on copper grids and analyzed using a Zeiss LIBRA 120 transmission electron microscope (Carl Zeiss, Oberkochen, Germany) operating at 120 kV.

**Adhesion Assay**

1 h post infection cells were washed three times with PBS to remove non adherent *C. albicans* cells and fixed using periodate-lysine-paraformaldehyde solution (PLP). After another washing step samples were blocked using Odyssey blocking solution for 1 h. *C. albicans* was then stained for another hour with rabbit-anti-*C. albicans* antibody (Acris, Germany). After five washes with 200 µl PBS supplemented with 0.1% Tween20 for 5 min, a goat-anti-rabbit 800CW secondary antibody solution was applied (Li-cor, USA) and incubated for 1 h. In the last washing step cells were washed five times with 200 µl PBST followed by one wash with PBS. Cells were kept dark in 100 µl PBS at 4°C until read-out. Quantification of fluorescence was performed using a Li-cor Odyssey® SA imaging system at 100 µm resolution and a focus offset of 3.0 mm. Fluorescence of the PBS-control was assumed to correlate to 100% adhesion, adhesion of the LGG-treated samples was calculated relative to the control.

**Invasion Assay and Hyphal Growth**

3 h post infections cells were rinsed three times with PBS and fixed for 2 min with PLP. To stain non-invasive growing parts of *C. albicans* rabbit-anti-*C. albicans* antibody (Acris, Germany) was added for 1 h. After three washes epithelial cells were permeabilized with 0.5% Triton-X 100 solution in PBS and washed again. Entire *C. albicans* cells (non-invasive and invasive parts) were then stained with human-anti-*C. albicans* antibody (Dr. Merck, Germany) for 1 h. After rinsing again with PBS dilution of secondary antibodys (both Dianova, Germany) in PBS were applied for 1 h, donkey-anti-rabbit-DyLight488 (green, detection of non-invasive parts) and donkey-anti-human-Cy3 (red, entire *C. albicans* cell). Cells were washed three times with PBS, cell nuclei were stained for 5 min with DAPI (Sigma Aldrich, Germany). After one final wash coverslips were mounted on slides, using Fluormount-G (SouthernBiotech, USA) and kept at 4°C until microscopic analysis.
