# Supplementary figures and images for: Antifungal defense of probiotic Lactobacillus rhamnosus GG is mediated by blocking adhesion and nutrient depletion
Source: PLoS One. 2017 Oct 12;12(10):e0184438. doi: 10.1371/journal.pone.0184438 (PMC5638248; doi:10.1371/journal.pone.0184438)

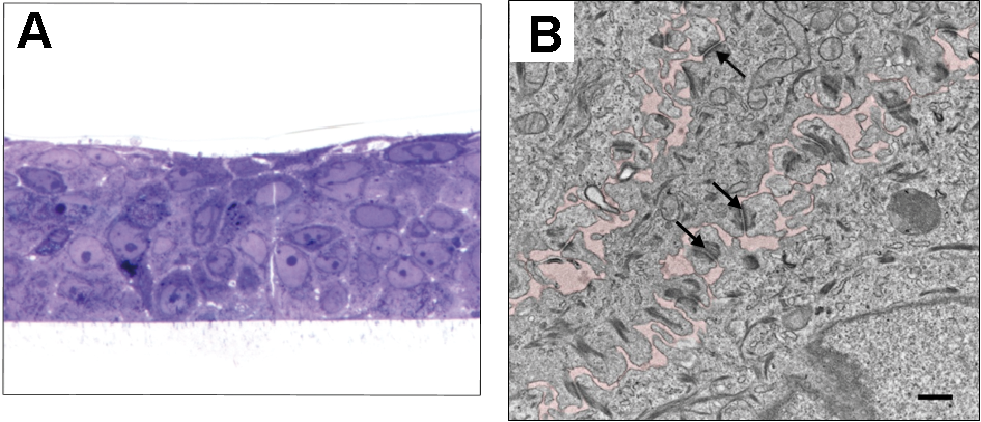

Supplement: S1 Fig — Reconstructed human oral epithelium (RHOE) at day 8 of cultivation. (A) Semi-thin section stained with toluidine blue and visualized by light microscopy at a 400-fold magnification. (B) Ultra-thin sections were contrasted with osmium and visualized by transmission electron microscopy. Arrows indicate desmosomes. Scale bar equates 0.5 μm. (TIF) [file pone.0184438.s002.tif]
