# Supplementary material for: Antifungal defense of probiotic Lactobacillus rhamnosus GG is mediated by blocking adhesion and nutrient depletion
Source: PLoS One. 2017 Oct 12;12(10):e0184438. doi: 10.1371/journal.pone.0184438 (PMC5638248; doi:10.1371/journal.pone.0184438)
Supplement: S1 Table — Summary of the different media used to generate three dimensional models of human oral mucosa. (DOC) [file pone.0184438.s003.doc]

**S1 Table.** Generation of three-dimensional mucosal models.

| d0 | d1 - d4 | d4 | d5 - d8 | d8 |
| --- | --- | --- | --- | --- |
| DMEM + 10% FBS;  KBM-Gold™ | KBM-Gold™ | KGM-Gold™ without antibiotics | KGM-Gold™ without antibiotics + 2mM CaCl_2_ | KGM-Gold™ without antibiotics |
