# Supplementary material for: Antifungal defense of probiotic Lactobacillus rhamnosus GG is mediated by blocking adhesion and nutrient depletion
Source: PLoS One. 2017 Oct 12;12(10):e0184438. doi: 10.1371/journal.pone.0184438 (PMC5638248; doi:10.1371/journal.pone.0184438)
Supplement: S3 Table — The upper panel shows the amount of glucose in the supernatants of differentially treated monolayers of TR146 keratinocytes. In some cases the medium was supplemented with glucose (5 mg/ml). The lower panel shows the amount of glucose in KGM-Gold™ medium incubated with LGG and or C. albicans. n = 3. (DOC) [file pone.0184438.s005.doc]

**S3 Table**. **Availability of glucose.**

| **TR146** | **0 h - 12 h** | **Supplement** | **12 h – 18 h** | **Glucose [µg/ml] ± SEM** |
| --- | --- | --- | --- | --- |
| yes | PBS  LGG  LGG  PBS  LGG  LGG | H_2_O  H_2_O  Glucose  H_2_O  H_2_O  Glucose | PBS  PBS  PBS  *C. albicans*  *C. albicans*  *C. albicans* | 985.4 ± 56.3  0.7 ± 0.1  1482.5 ± 79.8  437.6 ± 39.3  0.6 ± 0.0  908.3 ± 143.0 |
| no | PBS  LGG  PBS  LGG | n/a | PBS  PBS  *C. albicans*  *C. albicans* | 1884.13 ± 178.0  971.6 ± 113.2  910.6 ± 102.3  328.5 ± 112.7 |
