# Supplementary material for: Antifungal defense of probiotic Lactobacillus rhamnosus GG is mediated by blocking adhesion and nutrient depletion
Source: PLoS One. 2017 Oct 12;12(10):e0184438. doi: 10.1371/journal.pone.0184438 (PMC5638248; doi:10.1371/journal.pone.0184438)
Supplement: S4 Table — LGG (1.9 x 10^6^ CFU/ml) and C. albicans (1 x 10^6^ CFU/ml) were inoculated to KGM-Gold™ or MRS broth. OD600 was determined immediately and after 12 h incubation. pH was determined after 12 h incubation. To assess hyphal growth C. albicans was allowed to grow for 18 h. (DOC) [file pone.0184438.s006.doc]

**S4 Table. Differences between KGM-Gold™ medium and MRS medium.**

| **Parameter** | **Experimental condition** | **KGM-Gold™** | **MRS** |
| --- | --- | --- | --- |
| OD_600_  LGG | 0 h  12 h | 0.04 ± 0.003  0.10 ± 0.002 | 0.03 ± 0.003  7.89 ± 0.044 |
| OD_600_  *C. albicans* | 0 h  12 h | 0.24 ± 0.012  0.87 ± 0.132 | 0.23 ± 0.018  2.91 ± 0.135 |
| pH (after 12 h) | control  LGG  *C. albicans* | 7.5 ± 0  7.5 ± 0  7.5 ± 0 | 6 ± 0  4 ± 0  6 ± 0 |
| *Candida* hyphal growth | 18 h | yes | no |
