# Supplementary material for: Antifungal defense of probiotic Lactobacillus rhamnosus GG is mediated by blocking adhesion and nutrient depletion
Source: PLoS One. 2017 Oct 12;12(10):e0184438. doi: 10.1371/journal.pone.0184438 (PMC5638248; doi:10.1371/journal.pone.0184438)
Supplement: S5 Table — (DOC) [file pone.0184438.s007.doc]

**S5 Table. pH after 18 h in presence of human keratinocytes**

| **0 h - 12 h** | **12 h - 18 h** | **pH** |
| --- | --- | --- |
| PBS  LGG  PBS  LGG | PBS  PBS  *C. albicans*  *C. albicans* | 7.5 ± 0  7.0 ± 0  7.5 ± 0  7.0 ± 0 |
